# Regenerative Agriculture and Sustainable Plant Protection: Enhancing Resilience Through Natural Strategies

**DOI:** 10.3390/plants15010113

**Published:** 2025-12-31

**Authors:** Muhammad Ahmad Hassan, Ali Raza, Saba Bashir, Jueping Song, Shoukat Sajad, Ahsan Khan, Laraib Malik, Zoia Arshad Awan

**Affiliations:** 1 College of Resource and Environment, Anhui Agricultural University, Hefei 230036, China; ahmaduaf93@ahau.edu.cn (M.A.H.); khanahsanaau@gmail.com (A.K.); 2 Anhui Province Key Laboratory of Crop Integrated Pest Management, School of Plant Protection, Anhui Agricultural University, Hefei 230036, China; aliraza@stu.ahau.edu.cn (A.R.); songjueping@163.com (J.S.); laraib.aau163@gmail.com (L.M.); 3 Anhui Provincial Key Laboratory of Microbial Pest Control, Anhui Agricultural University, Hefei 230036, China; 4 School of Life Sciences, Anhui Agricultural University, Hefei 230036, China; sababashir079@gmail.com; 5 Lushan Botanical Garden, Chinese Academy of Sciences, 9 Zhiqing Road, Jiujiang 332900, China; sajadshoukat@cas.edu.cn; 6 Horticulture Development Department, Teagasc Food Research Centre, Ashtown, D15 KN3K Dublin, Ireland

**Keywords:** sustainable plant protection, regenerative agriculture, eco-friendly pest management, biodiversity and pest control, integrated pest management (IPM)

## Abstract

The world faces increasing food, environmental, and human security issues, primarily attributed to an overburdened agricultural sector struggling to keep pace with rising population and demand for food, energy, and fiber. Advances in food production and agriculture, especially with monoculture farming, have continued to meet these demands but at a high price regarding resource depletion and environmental devastation. This is especially severe in developing world areas with rural populations with thin resource margins. Regenerative agriculture has emerged as a solution to provide shielding for food production, ensure environmental protection, and promote social equity while addressing many of these issues. Regenerative agriculture food production aims to restore soils, forests, waterways, and the atmosphere and operate with lower offsite negative environmental and social impacts. This review discusses the fundamental principles and practices of sustainable plant protection for regenerative farming. It focuses on the role of biological and ecological processes, reduces non-renewable inputs, and aims to incorporate traditional ecological knowledge into pest control practices. It offers essential transition strategies, including critical changes from conventional integrated pest management (IPM) to agro-ecological crop protection, focusing on systemic approaches to design agroecosystems. It also reaffirms the importance of a vast diversity of pest control methods that are culturally, mechanistically, physically, and biologically appropriate for regenerative farming practices. Ultimately, the aim is to encourage ecological, economic, and social sustainability for the future of more resilient and controlled agricultural practices.

## 1. Introduction

Agriculture faces increasing pressure to meet the rising global demand for food, fiber, and energy while minimizing environmental degradation. Conventional farming systems, particularly those based on monoculture and intensive chemical inputs, have contributed significantly to yield gains; however, these advances have often come at the cost of soil degradation, biodiversity loss, pest resistance, and ecosystem instability [1,2]. Sustainable agriculture strives to produce the food we need while also ensuring that natural ecosystems remain intact as much as possible and minimize the harmful environmental impact. Although great strides have been made to improve crop productivity, these improvements generally come at a substantial ecological price. Notably, biotic stresses, such as pests, pathogens, and other negatively impacting organisms, still obstruct the path of agricultural productivity [3,4]. Plants are well known as sessile organisms, meaning that they cannot move away from these stresses, resulting in lower crop production, damaged ecosystems, and economic losses [5,6,7].

In recent years, there has been an increased understanding and availability of alternative agricultural methods, such as regenerative agriculture. Unlike conventional input-intensive systems, regenerative agriculture emphasizes soil structure, biological processes, and ecosystem functions to address interconnected challenges such as soil degradation, pest resistance, and climate variability [8,9,10]. The concept of regenerative agriculture was first articulated in the late 1970s and early 1980s, notably by Robert Rodale, who emphasized farming systems that not only sustain but actively regenerate soil health, biodiversity, and ecosystem functions [11]. Rodale’s vision extended beyond yield maintenance, proposing that agricultural systems should restore degraded soils, enhance biological processes, and improve environmental and human health simultaneously.

Since its early formulation, regenerative agriculture has evolved from a conceptual framework into a science-informed approach supported by advances in soil ecology, agroecology, and systems biology. Contemporary interpretations integrate principles such as minimal soil disturbance, permanent soil cover, crop diversification, and the promotion of beneficial biological interactions. These principles align closely with modern sustainability goals, positioning regenerative agriculture as a transformative strategy for addressing food security, climate resilience, and environmental degradation.

It is now a widely accepted approach to improving the food system that can simultaneously solve two major issues: the issues of food security and environmental degradation [12,13].

Plant protection lies at the core of regenerative agriculture. The focus is on minimizing the correct use of synthetic pesticides and fertilizers and promoting the natural enemies of pests, which have the potential to help plants grow. Since IPM is a part of this strategy, there is a growing concern about using agro ecological tactics as diverse pest control solutions, including biological, mechanical, non-chemical, and cultural measures [14,15]. These strategies reduce dependency on hazardous chemical inputs, enhance natural enemy populations, and contribute to long-term system stability and productivity.

This review synthesizes recent advances in regenerative plant protection, with a particular focus on agroecological and biological approaches to pest management. By critically examining current practices, emerging concepts, and future research directions, this article aims to highlight how regenerative plant protection can enhance agricultural sustainability, resilience, and productivity in the context of global environmental change.

## 2. Biodiversity-Driven Pest Management: A Natural Approach to Sustainable Agriculture

Regenerative agriculture is a comprehensive approach to farming that seeks to rebuild soil health and restore environmental quality through techniques that maximize the sustainable use of land, water, and vegetation. A symbiont relationship has developed between farming practices and natural ecosystems, creating high-quality water, improved land productivity, and higher biodiversity [16]. Biodiversity in regenerative agriculture should be understood in functional terms, where the presence of diverse predators, parasitoids, pollinators, soil fauna, and microbial communities enhances ecosystem services directly linked to pest regulation and plant resilience. In recent years, the rise of interest in regenerative agriculture can be characterized by its adoption through wide groups of actors (farmers, retailers, academics, policymakers, and even media), which have increasingly started to perceive regenerative agriculture as a model capable of transforming agricultural systems into sustainability [17].

The promotion of biodiversity as one of the corrosive elements in regenerative agriculture is vital for natural pest control. One of the main strategies to manage pest populations in agro and natural ecosystems is to use species navigation plants that attract beneficial insects, offer habitats for predators and pollinators, as well as cover crops and hedgerows [17,18]. Pest management approaches and biodiversity conservation have allowed agro-ecological measures to spread and offer promising and more sustainable alternatives to conventional chemical-based pest control strategies. The ratio of pest defenders is important in the study of the agroecosystem because it is considered to assess pest species in relation to their natural enemies [19,20]. Another piece of evidence is the research conducted on agroecosystems, especially organic tomatoes, which revealed that a number of these defensive organisms, including ladybugs, lacewing, and parasitoid wasps, are naturally occurring and in place of synthetic pesticides can control pest populations [21]. This underscores the need for setting up the right conditions for such beneficial bugs, which, among other things, assist in pest control as well as pollination and soil health

Floral resources provided by specific plant species are essential for attracting and sustaining these insects. *Centaurea cyanus* (cornflower), *Fagopyrum esculentum* (buckwheat), and Vicia sativa (vetch) serve as crucial nectar sources for widely distributed parasitoids, such as *Telenomus laeviceps*, which functions as a natural enemy of the pest *Mamestra brassicae* [22,23]. Non-crop plants demonstrate the capacity to support a greater diversity of pollinators, including bees, hoverflies, and butterflies, which are fundamental for promoting biodiversity and enhancing crop yield.

Another method for enhancing biodiversity is to establish native plant hedgerows along field boundaries. Alternative vegetation to traditional lawns can provide habitats and refuges for beneficial insects, as well as nesting sites for pollinators, such as solitary bees, and serve as wildlife movement corridors [24,25]. In numerous European countries, there have been increasing efforts to promote flowering strips within agricultural landscapes to sustain pollinators and natural pest controllers, particularly in highly cultivated areas [26,27].

In addition, crops that are sown between them are now increasingly recognized as contributing to biodiversity stimulation, natural pest control, etc. Plantings associated with non-crop plants provide habitats for beneficial insects, suppress weeds, eliminate soil erosion, and improve soil fertility, especially when legumes [28,29] are inter-seeded. Besides assessing soil health, cover crops serve as food reservoirs for predator and predator benefits, offering a continual supply of food that is beneficial, even during low pest populations [30,31].

Wildflower strips, cover crops, and hedge rows (together with thorn trees, soon yielding space to paw paws) provide enormous ecosystem benefits. Wind and water erosion effects are mitigated, they become habitats for other wildlife populations, and they increase the genetic heterogeneity of the fragmented landscapes [32,33]. Diversifying agricultural systems by incorporating these biodiversity-promoting practices will increase farmers’ use of chemical pesticides, increase ecosystem services, and develop more resilient agricultural systems that are able to adapt to changing environments. An approach beyond food security is additionally useful for long-term landscape and agrobiodiversity health in the long run. Sustainable practices that emulate this ecological strategy, such as hedgerows, cover crops, and wildflower strips, provide a solvent approach to mitigate the risk of economic loss to crops and maintain biodiversity in farming systems without relying on chemical solutions. This is a way forward in the face of the unfolding challenges of both food production and environmental conservation.

## 3. Soil Microbiome and Plant Defense: Strengthening Resilience Through Healthy Soils

The connection between soil vitality and plant vigor is clear, but healthy soils can do much more than produce superior plant growth and higher pest tolerance; they can also serve as the foundation for resilient agricultural systems. Healthy soil contains high amounts of crucial nutrients essential for plant growth as well as resilience to cope with stress [34,35,36]. It has been shown that the health of the soil is not only important for the growth of the plant but also helps alleviate the impact of pests and diseases. For instance, soil microbes and specific plant-associated members of the rhizosphere can stimulate plant innate immunity, thereby improving plant tolerance to herbivores and pathogens [37,38].

Soil microorganisms have been a significant area of recent research because of their impact on pest management. Soil-dwelling beneficial bacteria and fungi can serve as biological pest control agents, decreasing reliance on chemical pesticides. However, the results have been mixed with respect to the practical use of these microorganisms. For example, research on GM Bt rice has demonstrated that although Bt proteins do not have a deleterious effect on soil microorganisms, they may modify the composition of soil fungi, which can affect non-target organisms (NTOs) [39,40]. Furthermore, studies have shown that different concentrations and variations in environmental conditions can significantly influence the functioning of bio-pesticides, indicating a more nuanced approach to understanding the impact of bio-pesticides on these pests as well as beneficial organisms in soil [41,42].

Microorganisms such as mycorrhizal fungi are essential for enhancing soil quality and plant health [43]. Soil microorganisms enhance plant resistance by activating induced systemic resistance (ISR) and systemic acquired resistance (SAR), mediated by jasmonic acid-, ethylene-, and salicylic acid-dependent signaling pathways, thereby improving tolerance to pests and pathogens [44,45,46,47]. Key soil microorganisms involved in nutrient acquisition, induced resistance, and biological pest suppression, along with their documented impacts on plant health. Mycorrhizal fungi are important for facilitating nutrient uptake, especially phosphorus, which is limited to many agricultural soils. In addition, their symbiotic relationships with plants enhance nutrient availability and disease resistance by eliciting innate immune responses [48]. A comparative overview of conventional and regenerative agricultural systems, highlighting differences in soil health, pest management, biodiversity, water use, and climate impact, is provided in Table 1.

**Table 1:** Comparison of conventional agriculture vs. regenerative agriculture practices.

Aspect	Conventional Agriculture	Regenerative Agriculture	Impact on Sustainability
Soil Health	Soil erosion rates can reach 10–100 times higher than soil formation rates [49].	Regenerative practices can increase soil organic matter by 1–2% annually, improving water retention and carbon sequestration [50].	Improves soil structure, water retention, and carbon sequestration.
Pest Management	2.5 million tons of pesticides are used annually worldwide, leading to pest resistance and biodiversity loss [51].	Biological controls and IPM reduce pesticide use by 30–50% while maintaining crop yields [52].	Reduces chemical inputs, promotes natural pest predators, and enhances ecosystem resilience.
Biodiversity	Monoculture farming has led to a 75% decline in insect biomass over the past 30 years [53].	Polyculture and hedgerows can increase pollinator diversity by 50–70% [54].	Enhances pollination, natural pest control, and ecosystem stability.
Water Usage	Conventional irrigation wastes 30–60% of water due to inefficiency [55].	Drip irrigation and mulching reduce water use by 20–50% while improving crop yields [56].	Conserves water resources and reduce runoff pollution.
Climate Impact	Agriculture contributes 24% of global greenhouse gas emissions [57].	Regenerative practices can sequester 2–5 tons of carbon per hectare annually [50].	Mitigates climate change and improves long-term agricultural resilience.

**Figure 1:**
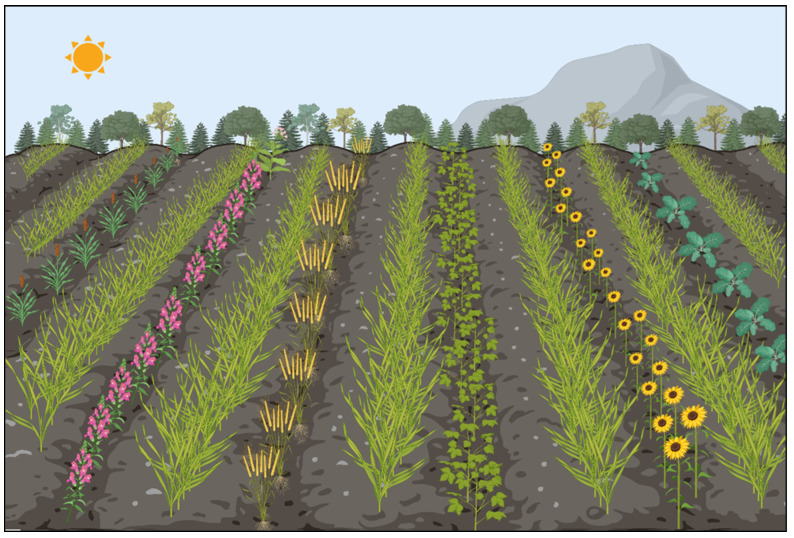
Schematic illustration of regenerative agriculture practices highlighting permanent soil cover, crop diversification, and the maintenance of living roots. These principles collectively enhance soil structure, promote belowground biological activity, support beneficial organisms, and improve agroecosystem resilience.

Another important aspect of the evergreen strategy is optimization of the soil structure [9]. The core principles of regenerative agriculture, including permanent soil cover, diversified cropping systems, and the maintenance of living roots, are schematically illustrated in Figure 1. Soils that are well-aerated, drained, and structured also encourage strong rooting systems, which are vital for plant health and stress resistance [58,59]. These non-commercial plants provide shelter for beneficial insects, suppress weeds, reduce soil erosion, and enhance soil fertility through nitrogen fixation, particularly when legumes are used [28,60]. Reduced tillage preserves soil structure and minimizes damage to soil organisms and exposure to weeds and pathogenic seeds [61,62]. Such practices contribute to soil ecosystems that are more resilient to pest pressure and, consequently, a decreased reliance on chemical inputs.

A simple approach to improve soil health and pest control is crop rotation. Farmers can also break pest cycles by planting crops with different nutrient requirements and pest susceptibilities [63,64]. In addition, polyculture schemes with diverse plants can increase biodiversity and inhibit pest settlement. By promoting positive relationships between plant species and their companion insects, polyculture enhances natural pest control and reduces pest pressure even more [65,66]. These more varied systems provide habitats for a wider variety of beneficial insects, which in turn can suppress pest populations and contribute to improved ecosystem function (Table 2).

**Table 2:** Soil microbiome and its role in plant defense.

Microorganism	Function	Impact on Plant Health	Examples
Mycorrhizal Fungi	Enhances nutrient uptake, especially phosphorus, and induces systemic resistance.	Increases plant growth by 20–50% and reduces disease incidence by 30–70% [67,68].	*Glomus* species.
Rhizobacteria	Produces compounds that stimulate plant immune responses (ISR and SAR).	Reduces pest damage by 40–60% and increases yield by 10–20% [69].	*Pseudomonas* and *Bacillus* species.
Nematophagous and Entomopathogenic Fungi	Preys on harmful nematodes in the soil.	Reduces nematode populations by 50–90% [70].	*Arthrobotrys*, *Metarhizium* species.
*Trichoderma* spp.	Antagonistic to soil-borne pathogens and promotes root growth.	Reduces fungal diseases by 30–70% and enhances nutrient uptake by 20–40% [71].	*Trichoderma harzianum*.
Nitrogen-Fixing Bacteria	Converts atmospheric nitrogen into plant-available forms.	Reduces synthetic fertilizer use by 30–50% [72].	*Rhizobium* and *Azospirillum* species.

Cover crops are also critical for building soil health and providing habitats for natural enemies of crops. Cover crops increase plant diversity [73,74] which, in turn, increases soil microbial biomass and activity, with downstream implications for nitrogen mineralization, carbon cycling, and improved soil structure. Some cover crops, such as Brassica species, have the potential to reduce the growth of pathogenic soil fungi and bacteria, in addition to promoting soil health and plant vigor [75].

Collectively, the soil-centered processes described above provide the mechanistic foundation for regenerative plant protection strategies. Enhanced soil structure, microbial diversity, and soil–plant interactions not only strengthen plant innate defense responses but also create conditions that favor preventive pest management approaches. Within this framework, healthy soils support the effectiveness of cultural control practices, promote biological pest suppression through natural enemies, and enhance the efficiency of physical and mechanical control strategies. The following subsections build upon this soil-based foundation to illustrate how regenerative plant protection translates soil microbiome-driven resilience into practical pest management solutions.

### 3.1. Cultural Control Methods in Regenerative Plant Protection

**Figure 2:**
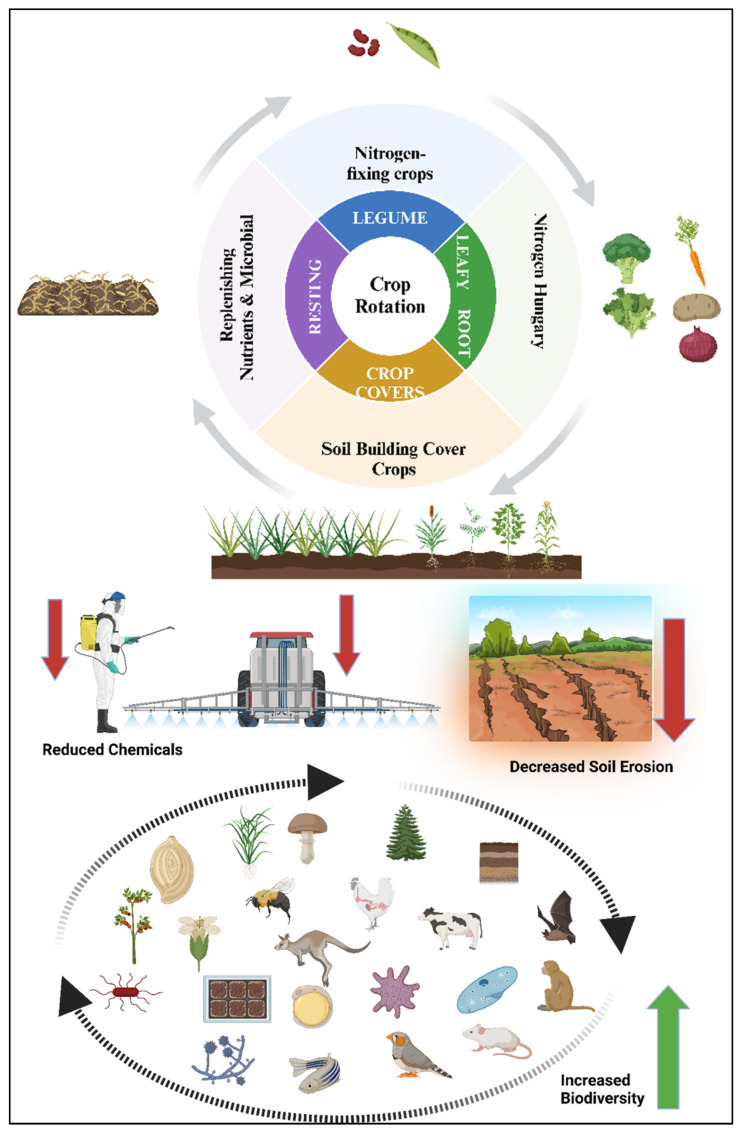
Conceptual framework illustrating the role of biodiversity in regenerative agriculture. Aboveground and belowground biodiversity contributes to ecosystem services essential for sustainable plant protection, including pest suppression by predators and parasitoids, enhanced pollination, improved soil structure, nutrient cycling, and microbe-mediated plant defense. Biodiversity is presented in terms of functional groups rather than individual species to emphasize ecological processes rather than taxonomic diversity.

Cultural control methods represent a preventive and system-oriented pillar of sustainable plant protection in regenerative agriculture [76,77]. Rather than targeting pests directly, these approaches modify cropping practices and agroecosystem conditions to reduce pest establishment, survival, and reproduction, while simultaneously enhancing soil health, biodiversity, and ecosystem resilience [78]. Cultural control aligns closely with regenerative principles by emphasizing process-based regulation, ecological balance, and long-term system stability (Figure 2).

#### 3.1.1. Crop Rotation as a Disruptive Ecological Strategy

Crop rotation remains one of the most effective cultural control strategies for managing insect pests, plant pathogens, and weeds in regenerative systems. By alternating crops with different functional traits and pest host status, rotation disrupts pest life cycles, reduces host continuity, and limits population carryover across seasons [79]. Beyond pest suppression, crop rotation enhances soil structure, nutrient cycling, and microbial diversity, which indirectly strengthens plant defense capacity. In regenerative farming, diversified rotations incorporating legumes, deep-rooted species, and cover crops reduce pest pressure while improving soil organic matter and biological activity, thereby reinforcing both above- and belowground resilience.

#### 3.1.2. Intercropping and Functional Crop Diversity

Intercropping and polyculture systems introduce spatial and temporal heterogeneity that interferes with pest host location, colonization, and feeding behavior. Increased plant diversity reduces monoculture-driven pest outbreaks by diluting host availability and altering microclimatic conditions unfavorable to pest development [80,81]. From a regenerative perspective, functional crop diversity supports trophic complexity, providing resources for natural enemies while reducing dependence on external pest control inputs. Intercropping systems also contribute to yield stability and risk reduction, key objectives in resilient agricultural design.

#### 3.1.3. Planting Time Adjustment and Phenological Escape

Manipulation of planting and harvesting dates enables crops to avoid synchronization with peak pest populations, a concept known as phenological escape. Early or delayed planting can significantly reduce pest damage without requiring direct intervention, particularly for pests with predictable seasonal dynamics [82,83]. In regenerative agriculture, such timing-based strategies complement ecological pest suppression by minimizing stress on crops during vulnerable growth stages, thereby improving the overall system efficiency and reducing the need for reactive control measures.

#### 3.1.4. Field Sanitation and Residue Management

Field sanitation plays a crucial role in reducing pest reservoirs within regenerative systems. The removal of infested plant material, volunteer crops, and unmanaged residues limits overwintering sites and early season pest establishment [84]. However, regenerative agriculture requires a balanced approach to sanitation and residue retention [85]. While excessive residue removal may undermine soil cover goals, strategic residue management such as selective removal or composting can suppress pests while preserving soil organic matter, microbial activity, and erosion control functions.

#### 3.1.5. Cover Cropping and Soil–Pest Interactions

Cover crops are multifunctional cultural tools that contribute to pest suppression through indirect ecological pathways [86]. By improving soil structure, enhancing microbial communities, and supporting natural enemies, cover crops strengthen plant health and resistance to pest attack. Certain cover crop species can also suppress pests directly through allelopathic effects or by disrupting pest habitat suitability [87]. In regenerative systems, cover cropping integrates pest management with soil regeneration, nutrient cycling, and carbon sequestration objectives.

#### 3.1.6. Habitat Manipulation and Landscape-Level Regulation

Habitat manipulation extends cultural control beyond the field scale to the agroecosystem and landscape levels [88]. The management of field margins, non-crop vegetation, hedgerows, and refuge areas increases ecological complexity and stabilizes predator–prey interactions. By enhancing habitat connectivity and resource availability for beneficial organisms, habitat manipulation reduces pest outbreaks while strengthening ecosystem services essential for regenerative agriculture, including pollination, biological control, and climate resilience [89].

Unlike chemical or curative interventions, cultural control methods function as anticipatory and system-level strategies. Their effectiveness increases when implemented collectively and integrated with biological, mechanical, and physical control approaches. In regenerative agriculture, cultural control is not a standalone tactic but a design principle guiding agroecosystem structure, function, and long-term sustainability.

### 3.2. Harnessing Nature: Biological Strategies for Pest Suppression

Biological control is an environmentally sustainable and effective pest management strategy that relies on natural enemies, including predators, parasitoids, and pathogens. This strategy reduces pest densities, which reduces crop damage, and can reduce the need for chemical treatments [90]. The three main strategies employed are importation, augmentation (in which pest control agents such as natural enemies are released to establish or enhance natural control processes), and conservation, all of which are widely perceived as ecologically appropriate, socially acceptable, and economically affordable. These strategies can be used alone or together as appropriate to the pest issue and the ag system [91,92].

### 3.3. Eco-Friendly Pest Control: Physical Barriers and Mechanical Strategies

Classic biological control is the introduction of a pest’s natural enemies from its native range to suppress pest populations in a new area. This strategy intends to use natural habitats to restore the equilibrium between pests and their natural predators in areas deficient in predators [92,93]. The success of this method relies on the identification of appropriate natural predators, determining the effects of these agents on pests in the native habitat, and controlling their introduction into the new habitat. Natural enemies have been introduced to the United States for the control of pests, such as the alfalfa weevil *Hypera postica* [94].

Therefore, the introduction of biological control agents must be regulated to minimize the risk of accidental introduction of other harmful organisms. Quarantine procedures are necessary to determine potential risks and confirm that only the organisms are intended to be released into the environment. This cautious approach is essential for the success of many biological control programs.

### 3.4. Botanical Pesticides in Regenerative Plant Protection

Botanical pesticides represent an important pillar of regenerative plant protection systems, offering environmentally compatible alternatives to synthetic pesticides while aligning with the ecological principles of soil health, biodiversity conservation, and reduced chemical dependency [95,96]. These products are derived from plant secondary metabolites that have evolved as natural defense compounds against herbivores and pathogens and have been used in traditional agriculture for several millennia. Although the widespread adoption of synthetic pesticides during the mid-20th century temporarily displaced botanical preparations, growing concerns over pesticide resistance, environmental contamination, and non-target toxicity have renewed scientific and practical interest in botanical pesticides within agroecological and regenerative farming frameworks [97].

Botanical pesticides encompass a broad and diverse group of preparations that can be classified into three major categories: (i) crude plant extracts and farm-made formulations, (ii) standardized commercial botanical insecticides, and (iii) essential oil-based products [98]. Crude extracts and decoctions, often prepared locally from readily available plants, are widely used in small-scale and low-input farming systems and remain particularly relevant in regenerative and organic agriculture [99]. These preparations typically act as repellents, feeding deterrents, or growth regulators rather than acute toxins, thereby reducing selection pressure for pest resistance.

Commercial botanical insecticides are generally produced from a limited number of well-studied plant species, including *Azadirachta indica* (neem), *Chrysanthemum cinerariifolium* (pyrethrum), *Nicotiana tabacum* (nicotine), *Derris* spp. (rotenone), and *Capsicum* spp. (capsaicin) [100,101]. Among these, neem-based products dominate the global market due to their broad spectrum of activity, multiple modes of action (antifeedant, growth regulator, oviposition deterrent), and comparatively low toxicity to non-target organisms. Importantly, the complex chemical composition of botanical pesticides, often involving synergistic mixtures of compounds, reduces the likelihood of resistance development compared with single-mode synthetic pesticides [101].

Essential oil-based botanical pesticides constitute a rapidly expanding subgroup. Oils derived from aromatic plants such as Thymus, Mentha, Cymbopogon, Eucalyptus, Rosmarinus, and Citrus species exhibit insecticidal, repellent, fumigant, and oviposition-deterring activities [102]. Their rapid biodegradability and low residue persistence make them particularly compatible with regenerative agriculture, although their volatility and short field persistence often require improved formulation strategies such as encapsulation or integration with other control measures.

Within regenerative plant protection, botanical pesticides are most effective when integrated with cultural, biological, and mechanical control strategies rather than used as stand-alone inputs. Their role is primarily preventive and suppressive, supporting ecological balance while minimizing disruption of beneficial insects, soil microbiota, and trophic interactions. Consequently, botanical pesticides should be viewed not as direct replacements for synthetic chemicals, but as adaptive tools embedded within diversified and resilient pest management systems. The major categories of botanical pesticides, their plant sources, active compounds, and functional roles in regenerative pest management systems are summarized in Table 3.

### 3.5. Augmentation of Biological Control: Enhancing Natural Enemy Populations

Augmentation works to enhance biological control by introducing additional natural enemies. This can be achieved by mass-producing and periodically releasing beneficial organisms, or through genetic enhancement to make them more efficacious [93]. The most prevalent strategy is mass rearing, in which insectaries rear abundant amounts of natural enemies released in pest-infested areas.

Augmentation is a well-known example in which *Encarsia formosa* (a parasitoid wasp) is exploited to manage *Trialeurodes vaporariorum* (greenhouse whitefly) populations in vegetable and floriculture crops [104]. This lady beetle is directly introduced into the crop as soon as early signs of whitefly pressure appear, which may counter the whitefly population before it reaches damaging levels. Augmentation is particularly valuable when natural enemies are scarce or when the response to pest outbreaks is slow.

## 4. Conservation of Natural Enemies: Enhancing Habitat and Resources

The conservation of natural enemies involves establishing and maintaining favorable conditions for the survival and reproduction of beneficial organisms. This can be achieved by adapting farming practices to ensure that natural enemies have access to sufficient food, shelter, and appropriate microclimates [105]. Maintaining alternate hosts, overwintering habitats, and a constant food supply for predators may supplement their efficacy in agricultural systems [106]. For instance, host plants and flowers that provide nectar and pollen for parasitoids and pollinators to thrive can help maintain their populations. As a result, hedgerows, cover crops, and wildflower strips are increasingly being integrated into agricultural landscapes to benefit these organisms [91]. In addition to food and shelter, these infrastructures act as corridors connecting crop fields for natural enemies to travel and enhance pest control.

### Challenges and Limitations of Biological Control

Although it has great potential, biological control is not without challenges, which have limited its widespread adoption. One significant problem is the relative slowness of biological control methods compared with chemical pesticides, which can lead to quick victories. The principal biological control strategies used in sustainable and regenerative plant protection, along with their advantages and limitations, are outlined in Table 4. As farmers (especially in very competitive agro-ecological market environments) look for solutions that will have the quickest and most favorable impact, this can make the transition to biological control methods a much less appealing option [107]. Additionally, biological control is often used to suppress, rather than eradicate, pest populations, which may not be an acceptable option for growers who want immediate pest-free produce.

Well-documented cases of biological control that have resulted in unintended consequences include the introduction of the cane toad to Australia to control the greyback cane beetle (Cossid) and the release of the harlequin ladybird (*Harmonia axyridis*) in Europe for the purpose of controlling aphids. In both instances, the introduced species became invasive, causing significant ecological damage [108,109].

**Table 4:** Biological control methods and their applications.

Method	Description	Advantages	Challenges
Importation	Introducing natural enemies from the pest’s native range.	Successful in 70% of cases for invasive pest control [110].	Risk of non-target effects and ecosystem disruption.
Augmentation	Releasing mass-reared natural enemies to enhance pest control.	Reduces pest populations by 50–80% in greenhouse crops [111].	High cost and labor-intensive.
Conservation	Modifying habitats to support natural enemy populations.	Increases natural enemy populations by 30–60% [112].	Requires knowledge of local ecosystems and species interactions.
Classical Biological Control	Establishing permanent populations of natural enemies in new environments.	Long-term pest suppression with 50–90% success rates [113].	Time-consuming and requires careful risk assessment.
Inundative Release	Releasing large numbers of natural enemies for immediate pest control.	Effective for short-term pest suppression in 80% of cases [114].	Expensive and may require repeated releases.

Furthermore, chemical pesticides may interfere with biological control strategies through the suppression or elimination of natural enemies. In addition to directly eradicating beneficial populations, they alter other habitats critical for the survival of these beneficial organisms, thereby reducing the efficacy of biological control [115,116]. Consequently, it is imperative that biological control be integrated into sustainable pest management while judiciously utilizing chemical inputs, as environmental conservation necessitates.

## 5. Plants Physical & Mechanical Protection

By emphasizing the continued significance of physical and mechanical control strategies in Integrated Pest Management (IPM) as the most efficacious and environmentally sound means of preventing pest damage to crops, the passage asserts that: these techniques encompass physical barriers, mechanical procedures, and ecological approaches to dynamically reduce insect populations below economic thresholds and minimize associated impacts, as opposed to the use of chemical compounds. As chemical pesticides have become increasingly restricted or undesirable in ecological or economic terms over recent decades, physical control strategies have been employed more extensively.

### 5.1. Insect Exclusion Screening

Insect exclusion screening is a commonly used physical control measure that has been proven to be extremely helpful in excluding insect pests from crops. This technique uses fine mesh screens or mosquito nets to remove pest species, such as whiteflies and fruit flies, from crops. The use of exclusion screens overcame the challenges imposed by whiteflies spreading viral diseases, which rendered open-field tomato production throughout the Mediterranean region impossible during late spring to fall. The addition of screens not only allows growers to lengthen the growing season, ensuring year-round tomato production, but is also a common and integral pest management strategy to mitigate losses in many cropping systems [117,118].

Exclusion screening is also used in orchards to keep pests out of the trees. This technique has been shown to be economically viable in crops, including peaches and nectarines, excluding damaging insect pests, such as the Mediterranean fruit fly, by netting, which has been shown to enhance both yield quantity and quality [119]. Similar strategies have been used to mitigate the spread of papaya dieback and disease vectors in banana plantations [120]. In apple orchards, such as physical barriers such as cellulose sheeting, are also employed to control weed species and insect pests, such as plum curculio and apple sawfly, that attack/develop fruitlets [121,122].

### 5.2. Difficulties and Drawbacks of Physical Barriers

Exclusion screening provides many advantages; however, it is not without its challenges. For instance, the accumulation of dirt in protective sheets can assist in weed growth, thus decreasing their potential of the method [123,124]. Furthermore, several of the materials commonly used as baseliners (e.g., cellulose sheeting) can degrade over time through the accumulation of water and plant debris and potentially interfere with their expected protective function(s). With this in mind, however, many types of physical barriers can be effective pest control tools in numerous situations, and their use is often combined with other pest management techniques.

#### 5.2.1. Specific Pest Species—Physical Control

For example, these have been shown to be effective against a range of pest species, including psyllids, aphids, spider mites, leafhoppers, and some Lepidoptera and beetle species [125,126]. Besides controlling arthropod pests, these techniques can also alleviate heat stress in trees and help to control filamentous fungal infections. Modified environment therapies (METs) such as fruit coatings and waxes were used to decrease the amount of oxygen and increase carbon dioxide in the fruit to combat tephritid fruit fly immatures [95]. Similarly, insect traps and coatings catch pests in the air by physically embedding them and immobilizing them, preventing them from completing their life cycle.

#### 5.2.2. Grow-Out Practices (Hand-Picking and Remove)

For smaller operations, hand picking and other manual pest control practices are viable and successful solutions. This method is particularly effective in places where there is ample labor and pest visibility. Handpicking has been used to remove pests, such as caterpillars (e.g., *Pieris brassicae* and *Papilio demoleus*) and other slow-moving insect larvae, which can be found in conspicuous locations. This approach has been successful in managing pests in small-scale settings including lawns, kitchen gardens, greenhouses, and small-scale tunnel farms [127]. For example, in cotton crops, removing infected flowers and larvae people often collect to treat the pink bollworm (*Pectinophora gossypiella*), which would help promote the killing of crops.

#### 5.2.3. Crop Protection and Barrier Methods

Another mechanical approach that shows acceptance is the use of physical barriers to prevent pests from damaging crops, in addition to screening and hand selection methods. Preventing fruit fly infestation by bagging individual fruits with paper or polythene bags can be effective, achieving 95% success rates [127,128]. These species serve as barriers for insects, which are common fruits (such as apples and peaches) and major crop land contributors.

In addition, in some areas of plantations, water-filled or insecticide-treated barriers are utilized to block insect migration between fields and protect crops including wheat and berseem from pests like armyworm larvae [127,129]. Thinner coverings that allow sunlight to pour in to protect young trees or crops can also help prevent insect infestation.

#### 5.2.4. Production of Apple with Insect Exclusion Netting

A new generation of insect exclusion netting has proven successful in controlling apple maggot flies and related pests. The introduction of netting systems in both France and Italy has been proven to reduce dependence on chemical pesticides by acting as a barrier against the major pest, the Codling moth (*Cydia pomonella*), in apple production [130,131]. These netting systems prevent moths from laying eggs on the fruit and diminish the number of larvae that can harm apples. While exclusion nets also protect crops from harmful pests, they limit access to beneficial insects such as ladybugs and syrphid flies, which are important players in supporting natural pest control services. Consequently, researchers are attempting to fine-tune these systems to find an equilibrium between safeguarding crops and conserving beneficial insect populations [132,133].

## 6. Green Chemistry: Plant-Derived Pest Control Solutions

This has also led to an increased interest in and demand for greener and non-toxic alternatives to synthetic pesticides because of the growing awareness of the harmful nature of chemical pesticides for human health and the environment. This has led to the development of organic and plant-based pest repellents as alternatives for pest management. Plant extracts and oils have been used to protect crops and gardens without the side effects of synthetic products.

### 6.1. Plant-Based Repellents and Efficacy

Plant-based repellents (as neem oil, lemongrass oil, eucalyptus oil, and catnip oil) have been shown to be effective against a broad spectrum of pests. For instance, neem oil is derived from the seeds of the neem tree (*Azadirachta indica*) and is recognized for its wide-ranging insecticidal effects. It disrupts the reproductive and feeding cycles of a range of pest species, including aphids, mealybugs, whiteflies, and red spider mites. Its active agent, azadirachtin, which is a natural growth regulator, impedes the growth process of bugs, thus making neem oil a universal agent for pest management [134,135].

It also acts as an insect repellent and pesticide due to citral in lemongrass oil. It is most effective against mosquitoes and other flying insects. Lemongrass oil can be mixed in sprays or with water to wash surfaces, creating a natural barrier against pests and providing antibacterial and anti-inflammatory benefits. Catnip oil, an essential oil produced by extracting Nepeta cataria, has been shown to be 10 times more repellent to mosquitoes and Aedes aegypti, a synthetic pesticide commonly used in commercial repellents [136,137].

### 6.2. Plants and Pests: The Battle of Chemical Defenses

The other half of the recipe consists of ingredients derived from plants that contain their own natural chemicals to protect against herbivores and other pests. Nitrogen compounds (alkaloids), terpenoids, phenolics, proteinase inhibitors, and growth regulators fall into five broad categories, including repellents, feeding deterrents, toxins, and growth regulators [138,139]. They protect plants against phytophagous insects and serve as effective repellents for blood-feeding insects, such as mosquitoes.

It has already been demonstrated in research that volatile chemicals released by plants at the time of herbivory, so-called “green leaf volatiles”, are extremely effective in repelling herbivorous insects. Compounds such as citronellal, geranyl acetate, and thujone are classified as volatiles that can interfere with the olfactory receptors of pests, such as mosquitoes and flies [140,141]. This evolutionary capacity has allowed plants to survive, despite the presence of herbivores.

### 6.3. Natural Deterrents: Garlic and Peppermint

Other effective plant-based repellents include garlic and peppermint oils, which are organic alternatives to chemical pesticides. Garlic, which is infamous for its strong smell, has been used as a natural insect deterrent for hundreds of years. Homemade garlic spray is made by blending fresh garlic cloves with water and straining the mixture before use. This repellent is particularly strong against beetles, aphids, and other herbivores ones [142].

Peppermint oil harvested from the Mentha piperita plant is another natural deterrent that works well. It works against ants, spiders, and rodents. Dilute peppermint oil with water in a spray and/or apply directly to pest entrances, such as doorways or windows. Due to its potent aromatic properties, peppermint is a promising natural substitute for chemical pesticides [143].

### 6.4. Eucalyptus Oil and Other Natural Substances

Another plant-based repellent that is widely used for repelling airborne insects, such as mosquitoes, flies, and other pests, is eucalyptus oil, which has a strong odor. However, by making the space uncomfortable for insects to live, it serves as an efficient, all-natural repellent when used through sprays or diffusers [144]. Besides repelling insects, eucalyptus oil has antifungal and anti-inflammatory properties, making it an even more attractive multi-use natural remedy.

### 6.5. Environmental Benefits and Sustainability

Not only effective, but organic and plant-based pest repellents are also sustainable. Plant-based repellents are more eco-friendly than chemical pesticides, which can kill beneficial organisms and adversely affect the ecosystem. These repellents are less persistent in the environment, thus reducing pollution and ecosystem damage. Therefore, in addition to repelling harmful pests, many plant-based repellents boost the vitality of the soil by supplying additional nutrients and aiding growth. For example, grape fruit oil is obtained from the peels of citrus fruits and contributes to the stimulation of plant growth and essential soil nutrients [136].

### 6.6. Economic and Environmental Benefits

Assessing the economic efficiency of sustainable pest management systems is essential for agricultural and environmental sustainability [145]. This reduces the use of chemical pesticides, which are known to harm ecosystems and human health. Sustainable pest management approaches, such as organic agriculture and integrated pest management (IPM), seek to improve the resilience of agricultural systems and reduce their environmental impacts [146]. Thus, implementing sustainable pest control methods might incur a higher investment and thus a higher cost at the onset; however, they frequently promote long-term financial benefits in the long run and therefore prove to be a financially desirable choice for farmers.

### 6.7. Long-Term Economic Advantages of Sustainable Practices

While it may cost more to implement alternative pest control solutions such as natural predators, crop rotation, or organic repellents, the return on investment as a sustainable solution over time is significant [147,148]. This is mainly because it reduces the need for costly chemical pesticides that become less effective over time as pests build resistance. Upon reducing chemical inputs, farmers no longer spend money on chemical pest control and can increase profitability in the long-term. Moreover, sustainability practices also improve soil health and conserve water and yield, which could lead to cost savings and increased agricultural productivity [149].

## 7. Environmental Benefits of Reducing Chemical Inputs

Chemical pesticides pose a significant environmental threat, as they can contaminate soil, water, and other species, and contribute to pesticide-resistant pests. Reducing the use of chemical prevention methods to prevent the increased incidence of resistance to chemical pesticides can be achieved through sustainable pest management interventions, such as the principles of Integrated Pest Management (IPM) and the use of organic repellents and bio-pesticides [150]. Integrating natural predators and beneficial insects into pest control measures can help protect key ecosystem services needed to bring crops to market like pollination. The combined economic and environmental advantages associated with sustainable pest management approaches are summarized in Table 5. There are also serious health risks to workers and communities that are exposed to excessive pesticide use. Exposure to pesticides causes both acute and chronic health problems such as respiratory problems, neurological effects, and increased cancer risk [151]. Adopting sustainable pest management practices can reduce the risk of pesticide use globally. This is especially important for those who work in the agricultural sector, as well as communities living near farms and near potential pesticide residue exposure.

**Table 5:** Economic and environmental benefits of sustainable pest management.

Aspect	Economic Benefits	Environmental Benefits	Long-Term Impact
Reduced Chemical Inputs	Saves $20–50 per hectare on pesticide costs [52].	Reduces soil and water contamination by 30–70%.	Improves soil health and biodiversity, leading to sustainable farming.
Biological Control	Reduces pest control costs by 40–60% [114].	Promotes natural pest predators and reduces pesticide resistance.	Enhances ecosystem resilience and reduces long-term pest control costs.
Soil Health	Increases crop yields by 10–20% [50].	Enhances carbon sequestration and water retention.	Mitigates climate change and improves agricultural productivity.
Biodiversity	Reduces crop losses by 20–40% through enhanced pollination [54].	Maintains ecosystem balance and reduces the risk of pest outbreaks.	Creates a more resilient and sustainable agricultural system.
Climate Resilience	Reduces vulnerability to extreme weather events and pest outbreaks.	Enhances ecosystem adaptability to climate change.	Ensure long-term food security and environmental sustainability.

### 7.1. Promoting Soil Health, Biodiversity, and Climate Resilience

Sustainable pest management is a part of the larger framework of regenerative agriculture, which focuses on soil health, biodiversity, and ecosystem integrity. Soil health is strengthened through organic farming and regenerative agriculture, which also enhances water retention, nutrient cycling, and plant growth. Maintaining a varied range of agricultural plants and animals also helps to support biodiversity. This biodiversity not only sustains good pest control but also enhances the resistance of farming systems to environmental stresses, including pests, pathogens, and diseases, as well as extreme events such as El Ninos.

Sustainable farming practices promote healthy ecosystems, enabling farming systems to adapt more effectively to climate change. These ecosystems tend to be more resilient: they are less vulnerable to the negative consequences of changing environmental conditions, such as drought, flooding, and temperature variations. Furthermore, regenerative practices also help with carbon sequestration, assisting in combating climate change by lowering the amount of greenhouse gases in the atmosphere and by storing additional carbon in the soil [152].

Both current farmers and consumers stand to gain from adopting a long-term perspective on sustainable agriculture, which will ensure the future availability of fertile soils and productive lands. These practices help improve the health of ecosystems and support biodiversity, creating a sustainable and resilient agricultural system that can meet the increasing demand for food worldwide. Representative biodiversity-based strategies that enhance pest suppression and ecosystem services in regenerative agriculture are presented in Table 6.

**Table 6:** Biodiversity-driven pest management strategies.

Strategy	Description	Benefits	Examples
Hedgerows	Planting native shrubs and trees along field edges.	Increases beneficial insect populations by 40–60% [25].	Cornflower, buckwheat, and vetch.
Cover Crops	Non-crop plants grow between main crop seasons.	Reduces weed biomass by 50–90% and improves soil nitrogen by 30–50% [153].	Legumes like clover and vetch.
Wildflower Strips	Strips of flowering plants within or around crop fields.	Increases pollinator abundance by 300% and pest control by 50% [26].	Sunflowers, marigolds, and lavender.
Polyculture	Growing multiple crop species together.	Reduces pest outbreaks by 30–60% compared to monocultures [154].	Intercropping maize with beans or squash.
Biological Control	Introduction or conservation of natural enemies (predators, parasitoids, etc.).	Reduces pest populations by 50–80% without chemical inputs [114].	Ladybugs, lacewings, and parasitoid wasps.

### 7.2. Overcoming Barriers and Advancing Sustainable Plant Protection

Several crucial issues need to be addressed to ensure the long-term sustainability of plant protection, because sustainable plant protection is an evolving field. Although notable progress has been made in developing sustainable pest management systems that reduce reliance on traditional chemical pesticide use, several barriers need to be addressed to facilitate their widespread adoption. This landscape may be important for understanding the challenges of the efficacy and scalability of sustainable pest control measures in a solution space that can be tailored to different agricultural systems.

### 7.3. Key Challenges in Sustainable Plant Protection

Scale is one of the greatest challenges in sustainable plant protection. Many sustainable pest management approaches work well, even with excellent results in small-scale farms or small experimental plots but transferring to large-scale commercial agriculture is often a complicated professional prospect. However, small-scale studies do not always scale to larger operations because of the differences in farm size, crop diversity, and pest pressures. This gap requires scalable strategies and technologies that can be effectively implemented in larger agricultural production systems, especially intensive agricultural systems [145].

Sustainable plant protection practices are often constrained by resources, traditional agricultural practices, and resistance to change. Farmers in conventional systems tend to be cautious about adopting new practices, both because they fear the risk of failure and because they require them to invest in training, equipment, and meaningfully changing other systems and processes. This hesitance will not be overcome by merely showing the long-term benefits of sustainable methods; it will also mean having to address financial and practical concerns [147].

One significant barrier is the known gaps in our understanding of the complex ecological interactions, pest behaviors, and longer-term impacts of various pest control treatments. Identifying these factors (as well as others) is critical for creating better pest management programs that will be effective and sustainable over the long-term. Controlling pests through genetic strategies is still in its nascent evolutionary stages and requires further research into pest evolution and how various pest eradication strategies affect the environment and unintended (non-target) species. Such knowledge is important for ensuring the enduring survival of sustainable plant protection [155,156].

## 8. Future Research Directions

The future is exciting, with new technologies providing promising avenues for efficient and effective pest management. This transformation involves precision agriculture, remote sensing, and data-driven approaches in the form of technologies, such as artificial intelligence (AI) and machine learning. These technologies can aid farmers in the real-time monitoring of pest populations, prediction of pest outbreaks, and more informed decision-making regarding the timing and means of pest control measures [145]. Not only could this drive biological, chemical, and cultural control strategies in a more targeted manner, reducing reliance on harmful inputs, but it could also drive biological control systems through more effective pest control.

AI- and machine learning-based decision support systems are being developed to analyze large datasets derived from field sensors, satellite imagery, drones, and weather stations. These systems can detect early pest infestations through image recognition, predict pest outbreaks based on climatic and phenological data, and recommend optimal intervention timings. For example, AI-powered image analysis has been successfully applied to identify insect damage, disease symptoms, and weed pressure at early stages, enabling targeted responses that reduce unnecessary pesticide applications [157]. In regenerative systems, such tools support preventive and threshold-based interventions consistent with Integrated Pest Management (IPM) principles.

Precision agriculture technologies further enhance plant protection by enabling spatially explicit management practices [158,159]. Variable-rate application systems allow biological agents, botanical pesticides, or mechanical interventions to be applied only where and when needed. Soil sensors and proximal sensing technologies can monitor soil moisture, nutrient availability, and microbial activity, indirectly informing pest risk assessments by linking soil health indicators with plant susceptibility [160]. These approaches reduce input use, minimize environmental disturbance, and strengthen the soil–plant–microbiome continuum central to regenerative agriculture.

New biotechnological approaches also hold significant promise for sustainable pest management. Advances in microbial biotechnology, including the formulation of multifunctional microbial consortia and next-generation bioinoculants, aim to enhance plant defense through induced systemic resistance (ISR), nutrient mobilization, and competitive exclusion of pathogens [161,162]. In parallel, RNA interference (RNAi)-based technologies are emerging as highly specific pest control tools, capable of targeting essential genes in pest species while minimizing non-target effects [163]. When carefully regulated and integrated into ecological frameworks, such approaches could complement regenerative pest management without disrupting beneficial organisms.

Plant breeding and genome-editing technologies, such as CRISPR/Cas-based systems, offer additional avenues for developing pest-resilient crop varieties [164]. Rather than focusing solely on resistance traits that rely on single genes, future research is increasingly directed toward enhancing quantitative resistance, root traits, and plant–microbe interactions that improve resilience under low-input and biologically driven systems. These traits are particularly relevant in regenerative agriculture, where crop performance depends on ecosystem functionality rather than chemical compensation.

Climate change remains a critical driver shaping future pest dynamics. Rising temperatures, altered precipitation patterns, and increased climate variability are expected to influence pest distribution, phenology, and population pressure. Future research should therefore prioritize adaptive pest management strategies that integrate predictive modeling, climate-resilient crop varieties, and flexible agroecological designs capable of responding to shifting pest pressures.

Future work must include biological control strategies that incorporate native predators, parasitoids, and beneficial microbes. These natural agents decrease the use of chemical pesticides and maintain the ecological balance. The investigation of native species as potential biological control agents is especially relevant because it could serve as a more sustainable and feasible alternative to the release of foreign species, which has the potential to upset local ecosystems [151]. These include experiments on the role of microbes in the soil environment and their interactions for complete health and pest suppression.

Climate change also plays a significant role as it affects pest dynamics, posing a challenge and an area for future research. When global climate changes, as global temperature increases and weather patterns become more irregular, pest populations will change [to adapt] according to [altering] environmental conditions. There is a need to research pest-resistant varieties of crops and pest-resistant cropping systems that can withstand these changes. Droughts and floods will influence pest behavior, migration patterns, and life cycles; therefore, developing adaptive pest management strategies capable of protecting crops in increasingly changing environments will depend upon understanding how this transition occurs [146].

### Policies and Cooperation for Effective Execution

The provision of farmer education and extension services is equally important in driving sustainable plant protection. Effective communication about why sustainable practices are important, combined with readily available training and ongoing support, will be critical in driving the widespread adoption of these practices. “Governments and policymakers have a role to play in supporting sustainable pest management through laws and policies”, which may incorporate subsidies for natural farming, tax breaks for the utilization of sustainable bug control techniques, and strict standards on pesticide utilization [165].

Market incentives, such as certification programs and increasing consumer demand for organic and sustainable products, can also incentivize farmers to adopt more environmentally friendly practices. Through policies that expand access to financial and market support for sustainable agriculture, governments and industry actors can help address the often overwhelming financial barriers that prevent farmers from adopting more sustainable practices.

Researchers, farmers, policymakers, and industry stakeholders are working together to enable knowledge transfer, resource sharing, and integrated approaches to overcome the barriers to sustainable plant protection systems. The long-term impact assessment of sustainable pest management practices through strong monitoring and evaluation systems is also important. These systems will help direct future research activities, provide information for policy decisions, and ensure that sustainable practices continue to evolve, informed by empirical data and effects [148].

## 9. Conclusions

Numerous contemporary agricultural challenges, including the optimization of water usage, restoration of degraded soils, preservation of biodiversity, and carbon sequestration, can be addressed through regenerative agriculture. Scientific evidence continues to accumulate in support of its benefits, and while the potential of regenerative practices for the future of agroecologies is transformative, there remains a need for comprehensive evidence demonstrating the effects of these practices within terrestrial or aquatic ecosystems. Regenerative farming is a movement that incorporates sustainable plant protection strategies into its core principles to maintain ecosystem health. This is achieved by farmers adopting a holistic approach that focuses on restoring soil health, biodiversity, and ecosystem resilience. The techniques employed include the diversification of crop systems, land cover and cover crops, crop rotation, utilization of natural predators, and biological controls, which collectively contribute to the increased resistance of agroecosystems to pests and diseases over time. A significant advantage of regenerative agriculture is the reduction in the use of synthetic pesticides and herbicides. Regenerative farming substantially reduces pesticide use, enhances soil health, aids in biodiversity conservation, and contributes to climate change mitigation. These approaches offer a more sustainable and resilient agricultural system capable of meeting the increasing demands of global food production without compromising environmental well-being. The implementation of sustainable plant protection strategies to support regenerative agriculture necessitates collaboration among farmers, researchers, policymakers, and consumers. Such collective efforts will be instrumental in creating an agricultural system that is economically viable and environmentally sustainable while also ensuring a lasting heritage for future generations. Through the adoption of regenerative farming practices, progress is being made towards a healthier planet and the establishment of secure food systems for the years to come.

## Figures and Tables

**Table 3:** Major groups of botanical pesticides used in regenerative and agroecological pest management.

Group	Plant Source	Active Compound	Action	Use in Regenerative Systems
Neem products	*Azadirachta indica*	Azadirachtin	Growth inhibition	Broad, low-resistance control
Pyrethrum	*Chrysanthemum cinerariifolium*	Pyrethrins	Neurotoxicity	Targeted insect control
Essential oils	*Thymus*, *Mentha*, *Cymbopogon*, *Eucalyptus*, *Citrus* spp.	Terpenoids	Repellent/fumigant	Short-term suppression
Alkaloid extracts	*Nicotiana tabacum*, *Capsicum* spp.	Nicotine, capsaicin	Deterrence	Localized use
Rotenoid plants	*Derris*, *Lonchocarpus* spp.	Rotenone	Respiration inhibition	Limited historical use

Note: Detailed information regarding the contents of Table 3 can be found in Yadav et al. (2022) [103].

## Data Availability

The original contributions presented in this study are included in the article. Further inquiries can be directed to the corresponding author.

## Abbreviations

IPM	Integrated pest management
NTOs	Non-target organisms
ISR	Induced systemic resistance
SAR	Systemic acquired resistance
METs	Modified environment therapies
AI	Artificial intelligence
